# Identification of risk factors of cystic periventricular leukomalacia in preterm infants

**DOI:** 10.3389/fped.2026.1758972

**Published:** 2026-04-15

**Authors:** Renée Lampe, Irina Sidorenko, Eva Lück, Andrey Kovtanyuk, Ursula Felderhoff-Müser, Marcus Krüger, Christian Brickmann

**Affiliations:** 1 Department of Orthopedics and Sport Orthopedics, TUM School of Medicine and Health, Markus Würth Professorship at Technical University of Munich, Technical University of Munich, TUM University Hospital, Research Unit for Paediatric Neuroorthopedics and Cerebral Palsy of the Buhl-Strohmaier Foundation, Munich, Germany; 2 Department of Mathematics, Technical University of Munich, TUM School of Computation, Information and Technology (CIT), Munich, Germany; 3 Department of Paediatrics I, Neonatology, Paediatric Intensive Care, Paediatric Infectious Diseases, Paediatric Neurology, Centre for Translational Neuro- and Behavioural Sciences, University of Duisburg-Essen, University Hospital Essen, Essen, Germany; 4 Clinic for Neonatology, Munich Clinic Harlaching & Schwabing, Munich, Germany; 5 Department of Pediatrics, TUM School of Medicine and Health, Kinderklinik Muenchen Schwabing, Technical University of Munich, TUM University Hospital, Munich, Germany

**Keywords:** cerebral tissue oxygenation, cerebralblood flow, extremely and very preterm infants, hypoxia, partial pressure of oxygen in brain tissue, periventricular leukomalacia, risk factors, white matter injury

## Abstract

**Introduction:**

The neonatal period in very preterm born infants is characterized by high vulnerability of the developing brain. Diffuse or cystic white matter injury—cystic periventricular leukomalacia (cPVL)—is one of the most serious complications leading to brain injury in preterm infants, associated with an increased risk of neurological impairments such as cerebral palsy.

**Methods:**

In the present study, parameters that may potentially influence the occurrence of cPVL are analyzed. The study is based on retrospective clinical data of 46 preterm infants with and without cPVL, including prenatal factors, birth-related information, neonatal diagnoses and routinely measured clinical monitoring data. The novelty of this work lies in the inclusion in analyses of cerebral blood flow (CBF), CBF fluctuations and partial pressure of oxygen in brain tissue (PtO_2_), calculated from routinely measured parameters using a mathematical model.

**Results:**

Statistical analyses revealed significant association between cPVL and a group of 5 clinical and 23 regularly measured parameters including mathematically calculated CBF, CBF fluctuations and PtO_2_. The obtained results may be useful for future risk assessment of cPVL at an early stage.

## 1 Introduction

Preterm birth is the most common cause of infant mortality and morbidity worldwide, and its prevalence has increased in most countries over the last 20 years. Thanks to advances in neonatology, the number of surviving infants is increasing, however, around 40% suffer from neurocognitive and motor consequences in the long term (1). Very preterm infants born before 32 weeks gestation (WG) are particularly at risk of early childhood brain injury often caused by intraventricular hemorrhage (IVH), diffuse and cystic white matter injury (dWMI, cWMI) (2). The most severe form of WMI, cystic periventricular leukomalacia (cPVL), is rare but often results in cerebral palsy, associated with impairments in motor skills, behavior, sensory perception and cognition (3, 4). Therefore, prevention of early childhood brain injury is of great relevance to future health of preterm infants (5).

The likelihood of developing any kind of WMI depends largely on gestational age (GA). Moreover, its pathogenesis is multifactorial and has both vascular and inflammatory causes, such as chorioamnionitis and postnatal infections (6, 7). In addition, arterial hypotension, hypocarbia (pCO_2_ < 35 mm Hg), hyperoxia (pO_2_ > 60 mm Hg), prolonged ventilation and hypoxic periods have been identified as independent and significant risk factors during the first 7 days of life (8). All these conditions lead to release of pro-inflammatory cytokines and oxidative stress.

In very preterm infants, the cerebral vascular system is still immature with limited ability to regulate blood flow. A combination of immature cerebral vasculature and impaired cerebrovascular autoregulation, described as pressure-passive cerebral circulation (3), leads to a disturbance in cerebral perfusion and a reduction in CBF, associated with the development of WMI (9). Severely reduced blood flow in the white matter correlates with an overall reduction in global CBF, which may serve as a potential marker for the risk of cerebral injury. A hypoxic state caused by reduced CBF can lead to irreversible neuronal damage, emphasizing the importance of stable cerebral perfusion in early development (9).

Since CBF is not routinely measured in clinical care, knowledge of accurate levels in preterm infants is limited. The previously developed mathematical model for calculating CBF (10, 11), which has been adapted to the preterm brain, was applied for calculation of CBF optimal limits for preterm infants using routinely measured clinical parameters (12). Additionally, it was coupled with a nonlinear model of cerebral oxygen transport (13) and applied to determine optimal limits of the partial pressure of oxygen (PtO_2_) in brain tissue (12).

Although the pathogenesis of WMI is relatively well understood, predicting of its occurrence in very preterm infants remains a challenge. The clinical diagnosis of cWMI is usually made on the basis of structural changes in the brain detected by serial cranial ultrasound (cUS) (14). Magnetic resonance imaging (MRI) at term equivalent age (TEA) provides the ability to diagnose more subtle changes in white matter such as dWMI, but availability is often limited in clinical routine (15). However, the tissue damage itself may develop for days or even weeks before obvious structural changes on imaging. Therefore, research efforts are focused on developing predictive models to identify infants at risk of WMI.

The large number of clinical parameters, routinely monitored in the neonatal intensive care unit (NICU), provides a rich database. Computational intelligence techniques, such as multivariate logistic regression analysis and machine learning algorithms, are widely used methods for feature selection and classification, decision tree generation, and risk score construction. Previous machine learning and statistical studies (16, 17) have shown that combination of clinical parameters and calculated CBF in multivariate regression model predicted IVH risk.

The aim of current study is to analyze regularly monitored clinical peri- and postnatal information as well as calculated CBF and PtO_2_ on the basis of retrospective clinical data from 46 preterm infants in order to determine the risk factors of cPVL (diagnosed using routine cUS).

## 2 Material and methods

### 2.1 Patient cohort

The present work is a retrospective analysis of routinely measured clinical parameters that may lead to cPVL with particular attention to mathematically calculated CBF and PtO_2_. For this purpose, clinical records during first two weeks of life were collected from 46 infants with gestational age between 22 + 4 and 33 + 4 WG who were treated after birth at two Level-III perinatology centers in Germany. The cPVL was diagnosed by cUS, routinely performed on day 1st, 3rd, 7th, 14th and 28th of life. Additional examinations were also carried out if risk factors in the first days of life increased the likelihood of cPVL occurring (e.g., arterial hypotension requiring catecholamines). Infants diagnosed with and those without cPVL are referred here as affected and control groups, respectively.

Medical information was retrospectively extracted from patient charts. Regularly monitored clinical parameters were collected during first two weeks after birth and divided into 5 groups (Table 1): 5 prenatal diagnoses (group G1), 10 birth-related parameters (group G2), 11 infant diagnoses (group G3), 34 regularly recorded clinical parameters (group G4). In addition, numerically calculated CBF, CBF fluctuations and PtO_2_ were included in statistical analyses as independent parameters (group G5). In the entire cohort, no cases of feto-fetal transfusion syndrome, disseminated intravascular coagulation, cholestasis or congenital malformations were observed.

**Table 1:** Regularly monitored clinical parameters used for statistical analyses.

Group	Parameters
G1: prenatal diagnoses	*in vitro* fertilisation (IVF), preeclampsia/EPH-gestosis, intrauterine growth restriction (IUGR), preterm premature rupture of membranes (PPROM), chorioamnionitis/amniotic infection syndrome (AIS)
G2: birth related parameters	gestational age (GA), birth weight (BW), birth mode (spontaneous delivery or surgical), sex, multiple birth, APGAR (1, 5 and 10 min), umbilical cord pH, surfactant administration
G3: infant diagnoses	sepsis, intraventricular hemorrhage (IVH, grades I-IV), respiratory distress syndrome (RDS, grads I-III), bronchopulmonary dysplasia (BPD, all grades), pulmonary hemorrhage, acidosis (respiratory, metabolic, or both), focal/spontaneous intestinal perforation (FIP/SIP), necrotizing enterocolitis (NEC), thrombocytopenia, erythrocyte transfusion, retinopathy of prematurity (ROP, all grads)
G4: regularly recorded clinical parameters	Blood gas analysis: carbon dioxide partial pressure (pCO_2_), base excess (BE), pH, hydrogencarbonate (HCO_3_^−^)
	Oxygen supply parameters: oxygen partial pressure (pO_2_), arterial oxygen saturation (SaO₂), transcutaneous oxygen saturation (SpO₂), maximum oxygen demand, duration of continuous positive airway pressure (CPAP)/High Flow, duration of mechanical intubation.
	Cardiovascular parameters: systolic pressure (SYS), diastolic pressure (DIA), mean arterial pressure (MAD), heart rate, respiratory rate
	Inflammation parameters: C-reactive protein (CRP), interleukin-6, leukocyte counts, lymphocyte counts, body temperature
	Hemostatic parameters: hematocrit (Ht), hemoglobin (Hb), erythrocyte count, thrombocyte count, partial thromboplastin time (PTT), international normalized ratio (INR), Quick, fibrinogen
	Metabolic parameters: potassium (K), calcium (Ca), sodium (Na), blood sugar, lactate, bilirubin
G5: numerically calculated parameters	Cerebral blood flow (CBF), CBF fluctuations (CBF-CBF_mean_), partial pressure of oxygen in brain tissue (PtO_2_)

### 2.2 Calculation of CBF

The mathematical model for CBF calculations is based on a hierarchical cerebrovascular model for the adult brain (18), previously adapted to the particularities of the immature brain of preterm infants (10, 11). In the model, the vascular system is simulated as 19 levels with parallel connected vessels, whose number, length and diameter depend on gestational age. The vascular system of the germinal matrix is included as an additional parallel capillary compartment, whose size also depends on gestational age (19). The CBF is calculated as the ratio between the cerebral perfusion pressure and the cerebrovascular resistance. Perfusion pressure is calculated as the difference between MAP and intracranial pressure. The latter is set at 5 mmHg for all patients, which corresponds to the mean values of 4–6.5 mmHg (20) and 5.1 mmHg (21) measured in infants.

The resistance of the cerebral vascular system is composed of the resistances of the individual vessels, which react to changes in the physiological parameters MAP, pO₂ and pCO₂ by the corresponding change in vessel diameter (vasoconstriction and vasodilation) (11, 22). The mathematical model accounts also for the dependence of blood viscosity on the Ht level (23, 24) and on the presence of solid, randomly oriented particles (red blood cells) in a viscous medium (25). In addition to the CBF value, we calculated its variation CBF-CBF_mean_ around the normal value CBF_mean_, which was determined in our previous study (11) as the fitting curve of the mean values at each week of gestation of healthy preterm infants.

### 2.3 Calculation of PtO_2_

The previously developed mathematical model for PtO_2_ calculation (13) describes the oxygen diffusion within the cylindrical region of a single capillary vessel, which corresponds to the Krogh tissue cylinder (26, 27). In the model, the length and diameter of the cerebral capillary are set at 60 μm and 5.6 μm, respectively (11). Radial distance of the Krogh tissue cylinder is set to 30 μm from the capillary wall, which corresponds to about half the average extravascular distance between the capillaries (27). Taking axis symmetry into account, the problem is solved using a numerical algorithm based on the finite element method (FEM) and implemented in the FreeFEM++ software (13, 28). We calculate the physiological lower “hypoxic” PtO_2_ limit for cells located in the so-called “lethal corner” near the boundary of the tissue cylinder at the venule end of the capillary (for numerical purposes, a distance of 5 µm from the boundary of the calculation area is set), representing the area with the lowest oxygen content (26, 29).

### 2.4 Statistical methods

In our study, the cPVL diagnosis plays a role of response variable, which has a binary type. The predictive variables (predictors) are parameters listed in Table 1. To determine cPVL risk factors, a univariate analysis is performed using the two-sided Wilcoxon rank sum test for continuous parameters and the Fisher exact test for categorical parameters (30). A significance level of 5%, which corresponds to a *p*-value of 0.05, was defined as the threshold for significant association between cPVL and predictors. The statistical analyses was performed using official MATLAB24b software.

## 3 Results

Tables 2, 3 contain the descriptive statistics with a total number of 46 patients (mean GA 27.75 ± 2.84, mean BW 1,049 ± 354), with 25 in the affected group (with cPVL) and 21 in the control group (without cPVL). The statistical characteristics of clinical parameters of the study cohort are shown in Table 2 and medical diagnoses in Table 3.

**Table 2:** Descriptive statistics of prenatal and birth parameters in the entire cohort.

Parameter	Patient cohort *n* = 46 (100%)		
	min	max	mean ± std
GA [weeks]	22 + 4	33 + 4	27.75 ± 2.84
BW [g]	520	1,950	1,049 ± 354
APGAR 1 min	1	9	4.98 ± 2.37
APGAR 5 min	1	10	6.72 ± 1.89
APGAR 10 min	3	10	8.17 ± 1.35
Umbilical cord pH	6	7.47	7.28 ± 0.25
Male sex	29 (63.0%)		
Multiple pregnancy	15 (32.6%)		
Spontaneous delivery	6 (13.0%)		
Surfactant administration	40 (86.9%)		

**Table 3:** Number and percentage of medical diagnoses in the entire cohort.

Diagnosis	Patient cohort
	*n* = 46 (100%)
Prenatal diagnosis	
IVF	6 (13.0%)
AIS	33 (71.7%)
PPROM	27 (58.7%)
Preeclampsia/EPH-gestosis	9 (19.6%)
IUGR	6 (13.0%)
Infant diagnosis	
IVH	19 (41.3%)
RDS	36 (78.3%)
FIP/SIP	6 (13.0%)
ROP	31 (67.4%)
NEC	9 (19.6%)
Sepsis	16 (34.8%)
BPD	15 (32.6%)
Pulmonary hemorrhage	5 (10.9%)
Acidosis (respiratory, metabolic, or both)	13 (28.3%)
Erythrocyte transfusion	28 (60.9%)
Thrombocytopenia	8 (17.4%)

Patient groups with and without cPVL were matched according to gestational age and birth weight. Thus, these parameters had statistically equal (*p*-value > 0.05) mean values and standard deviations (Table 4). Therefore, these parameters are not analysed further as cPVL risk parameters.

**Table 4:** Comparison of gestational age and birth weight for affected and control groups.

Parameter	with cPVL			without cPVL			*p*-value
	*n* = 25			*n* = 21			
	min	max	mean ± std	min	max	mean ± std	
Gestational age [WG]	22 + 4	33 + 4	27.67 ± 3.21	23 + 5	31 + 5	27.85 ± 2.39	0.724
Birth weight [g]	520	1,600	1,012 ± 355	550	1,950	1,093 ± 356	0.552

The statistical comparison of prenatal parameters (group G1), birth-related parameters (group G2), and infant diagnoses (group G3) in patients with and without cPVL is summarized in Table 5. For four diagnoses, namely IVH, respiratory distress syndrome (RDS), focal/spontaneous intestinal perforation (FIP/SIP) and retinopathy of prematurity (ROP), a statistically significant association with cPVL was detected. A comparison between the date of IVH and cPVL diagnosis (Table 6) shows that in 10 of 13 (77%) patients, who had both diagnoses, IVH was diagnosed earlier than cPVL (marked in bold). Thus, IVH, RDS and FIP/SIP can be considered as potential cPVL risk parameters.

**Table 5:** Statistical comparison of prenatal parameters (G1), birth related parameters (G2), and infant diagnoses (G3).

Parameter	with cPVL			without cPVL			*p*-value
	*n* = 25 (100%)			*n* = 21 (100%)			
	min	max	mean ± std	min	max	mean ± std	
Birth related parameters (G2)							
APGAR 1 min	1	9	4.76 ± 2.15	1	9	5.24 ± 2.64	0.423
APGAR 5 min	3	9	6.44 ± 1.71	1	10	7.05 ± 2.09	0.166
APGAR 10 min	3	9	8.00 ± 1.50	5	10	8.38 ± 1.16	0.447
Umbilical cord pH	6	7.47	7.24 ± 0.32	7.17	7.42	7.34 ± 0.07	0.397
Male sex	16 (64.0%)			13 (62.0%)			1
Multiple birth	9 (36.0%)			6 (28.6%)			0.75
Spontaneous delivery	3 (12.0%)			3 (14.3%)			1
Surfactant administration	22 (88.0%)			18 (85.0%)			1
Prenatal diagnoses (G1)							
IVF	2 (8.0%)			4 (19.05%)			0.389
AIS	17 (68.0%)			16 (76.19%)			0.744
PPROM	12 (48.0%)			15 (71.43%)			0.139
Preeclampsia/EPH-gestosis	6 (24.0%)			3 (14.29%)			0.478
IUGR	4 (16.0%)			2 (9.52%)			0.673
Infant diagnoses (G3)							
**IVH**	**15** (**60.0%)**			**4** (**19.05%)**			**0**.**0071**
**FIP/SIP**	**6** (**24%)**			**0** (**0%)**			**0**.**0247**
**ROP**	**13** (**52.0%)**			**18** (**85.71%)**			**0**.**0260**
**RDS**	**23** (**92.0%)**			**13** (**61.90%)**			**0**.**0282**
NEC	7 (28.0%)			2 (9.52%)			0.151
Sepsis	10 (40.0%)			6 (28.57%)			0.538
BPD	9 (36.0%)			6 (28.57%)			0.754
Pulmonary hemorrhage	4 (16.0%)			1 (4.76%) 0.357			
Acidosis (respiratory, metabolic, or both)	9 (36.0%)			4 (19.05%)			0.325
Erythrocyte transfusion	18 (72.0%)			10 (47.62%)			0.132
Thrombocytopenia	6 (24.0%)			2 (9.52%)			0.259

Significant parameters with *p*-value < 0.05 are marked in bold.

**Table 6:** Timing of cPVL and IVH diagnoses for patients with.

Day of detection	Patient 1	Patient 2	Patient 3	Patient 4	Patient 5	Patient 6	Patient 7	Patient 8	Patient 9	Patient 10	Patient 11	Patient 12	Patient 13
Patient's number	1	2	3	4	5	6	7	8	9	10	11	12	13
Day of IVH detection	2	4	4	5	3	5	1	3	1	13	3	3	1
Day of cPVL detection	14	14	44	43	3	14	8	61	26	30	3	21	1

The statistical comparison of routinely measured and numerically calculated parameters (groups G4 and G5) in the patient cohort is summarized in Table 7. A statistically significant association with cPVL was found for 21 parameters (marked in bold). Important to note that all calculated parameters, namely CBF, CBF fluctuations (CBF-CBF_mean_) and PtO_2_, show a significant association with cPVL. In addition, a moderate (*p*-value < 0.1) association with cPVL was found for 3 parameters (marked in italic). These 24 parameters can be considered as potential cPVL risk parameters.

**Table 7:** Statistical comparison of regularly measured (G4) and calculated parameters (G5).

Parameter	with cPVL			without cPVL			*p*-value
	*n* = 25 (100%)			*n* = 21 (100%)			
	min	max	mean ± std	min	max	mean ± std	
**pH**	**6**.**66**	**7.53**	**7.28** ± **0.10**	**6**.**95**	**7.53**	**7.33** ± **0.08**	**<0**.**0001**
**HCO₃⁻**	**9**.**4**	**30.6**	**20.03** ± **3.39**	**13**	**37.6**	**21.29** ± **2.62**	**<0**.**0001**
**BE**	**−22**.**6**	**5.5**	**−6.07** **±** **3.96**	**−42**	**7.1**	**−4.4 5** **±** **3.81**	**<0**.**0001**
**Heart rate**	**10**	**264**	**161.5** ± **16.71**	**36**	**291**	**159.4** ± **14.94**	**<0**.**0001**
**SYS**	**16**	**96**	**51.42** ± **11.43**	**26**	**97**	**54.38** ± **11.24**	**<0**.**0001**
**MAD**	**20**	**82**	**39.99** ± **9.30**	**19**	**84**	**41.28** ± **9.34**	**<0**.**0001**
Thrombocyte count	**23**	**626**	**155.97** ± **105.7**	**33**.**2**	**953**	**237.12** ± **147.41**	**<0**.**0001**
**Blood glucose**	**1**.**02**	**356**	**131.51** ± **60.18**	**1**.**35**	**314**	**112.61** ± **44.03**	**<0**.**0001**
**SpO_2_ [%]**	**31**	**100**	**94.81** ± **4.15**	**48**	**100**	**95.69** ± **3.24**	**<0**.**0001**
**CBF-CBF_mean_**	**−10**.**73**	**55.66**	**1.22** ± **7.42**	**−11**.**57**	**44.83**	**−0.12** **±** **5.73**	**<0**.**0001**
**PtO_2_**	**2**.**85**	**57.79**	**17.16** ± **8.17**	**2**.**58**	**52.78**	**18.09** ± **7.15**	**0**.**0001**
**Ht [%]**	**19**.**4**	**65**	**41.79** ± **7.22**	**26**	**64.5**	**43.64** ± **6.71**	**0**.**0001**
**Hb**	**4**.**9**	**49.5**	**14.26** ± **2.82**	**8**.**7**	**39.4**	**14.88** ± **2.56**	**0**.**0005**
**DIA**	**12**	**75**	**31.79** ± **8.97**	**10**	**90**	**32.89** ± **9.53**	**0**.**0005**
**pCO_2_**	**20**.**7**	**96**	**44.27** ± **10.50**	**21**.**3**	**99.1**	**41.91** ± **9.24**	**0**.**0006**
**Lymphocyte count**	**0**.**44**	**11**	**2.81** ± **1.83**	**1**.**03**	**32**	**6.90** ± **8.49**	**0**.**0032**
**CBF**	**3**.**08**	**70.11**	**14.02** ± **7.68**	**2**.**84**	**57.63**	**13.29** ± **6.08**	**0**.**0009**
**Potassium**	**2**.**9**	**8**	**4.39** ± **0.84**	**2**.**4**	**7.48**	**4.54** ± **0.80**	**0**.**0081**
**CRP**	**0**.**09**	**223.1**	**12.39** ± **27.21**	**0**.**19**	**71.9**	**6.03** ± **12.28**	**0**.**0237**
**Intubation duration [days]**	**0**	**57**	**15.58** ± **17.59**	**0**	**34**	**5.14** ± **9.21**	**0**.**0430**
**Calcium**	**0**.**86**	**3.4**	**1.4** ± **0.39**	**0**.**85**	**2.8**	**1.65** ± **0.35**	**0**.**0466**
*Sodium*	*121*	*160*	*140.08* ± *6.23*	*124*	*158*	*139.35* ± *5.09*	*0*.*0535*
*Respiratory rate*	*16*	*99*	*51.85* ± *11.50*	*20*	*172*	*52.38* ± *13.43*	*0*.*0548*
*pO_2_*	*23*	*110.3*	*53.80* ± *11.01*	*21*.*3*	*102*	*55.03* ± *9.57*	*0*.*0904*
Lactate	0.38	18.4	2.22 ± 1.93	0.52	78	1.87 ± 3.97	0.1135
Bilirubin	0.3	40	5.87 ± 2.62	0.9	13.5	5.63 ± 2.17	0.1377
Maximum oxygen demand [%]	25	100	76.65 ± 30.51	21	100	65.28 ± 27.89	0.1760
Interleukin 6	0.2	15,660	484.2 ± 2,043.2	1.1	4,775	148.63 ± 587.41	0.1839
Body temperature	34.8	38.8	37.13 ± 0.40	34.8	38.8	37.12 ± 0.37	0.1995
SaO_2_ [%]	51.4	99.4	87.22 ± 9.38	55	99.1	96.12 ± 8.65	0.2308
Quick	22	127	68.44 ± 21.99	51	126	82.67 ± 38.84	0.3083
PTT	32	181	79 ± 37.91	47	62	56.33 ± 8.14	0.3119
Erythrocyte count	1.9	5.4	4.11 ± 0.6	2.5	6.1	4.17 ± 0.64	0.4235
INR	0.9	3.2	1.34 ± 0.39	0.9	1.4	1.17 ± 0.25	0.4662
Leukocyte count	1.36	55.9	12.62 ± 10.09	1.5	41.4	12.94 ± 8.11	0.7834
Fibrinogen	3.1	491	177.13 ± 135.16	144	251	197.5 ± 75.66	0.8350
CPAP/High Flow [days]	1	120	36.92 ± 32.74	2	61	33.76 ± 19.22	1

Significant (*p*-value < 0.05) and moderate (*p*-value < 0.1) parameters are marked in bold and italic.

## 4 Discussion

In order to identify potential correlations with the occurrence of cPVL, a cohort of 46 preterm infants with and without cPVL was analyzed with regard to 5 prenatal diagnoses (group G1), 10 birth-related parameters (group G2), 11 infant diagnoses (group G3) and 33 regularly measured parameters (group G4). In addition, numerically calculated CBF, CBF fluctuations and PtO_2_ (group G5) were included in statistical analyses as independent parameters. For the calculation of CBF and PtO₂, the following factors important for early brain development were included in the mathematical model: 1) anatomical: underdeveloped and vulnerable cerebral vessels in the germinal matrix; 2) hemodynamic: disturbed cerebrovascular autoregulation leading to unstable CBF (4).

### 4.1 Association of cPVL with infant diagnoses

Statistical analysis of the parameters of groups G1–G3 (Table 5) revealed four infant diagnoses significantly associated with cPVL. These included IVH, RDS, FIP/SIP and ROP. The significant association between IVH (all grades) and cPVL detected in our study is consistent with previous findings (31), suggesting that IVH may disrupt the maturation and myelination of oligodendrocytes in the periventricular white matter through ischemic mechanisms. Similarly, Grant et al. (32) found that cPVL frequently coexisted with IVH, and this combination was associated with worse neurological outcomes. Timing analysis (Table 6) demonstrated that IVH preceded the diagnosis of cPVL in 77% of patients, supporting Volpe (5), who noted that IVH often precedes periventricular lesions due to venous outflow obstruction.

It was observed that RDS occurred more often in infants with cPVL. The cause of RDS is multifactorial. Often, initial inflammation can cause RDS, which then affects the brain. In addition, late-onset of RDS can be a manifestation of a serious disorder that often affects not only the respiratory system but also, very quickly, the circulatory system in this group of patients. These in turn promote the development of cPVL. Treatment typically involves ventilation to maintain oxygenation (33). The link between RDS and cPVL may stem from impaired lung function and resulting hypoxia, causing arterial oxygen and carbon dioxide levels to fluctuate. Such a clinical picture disrupts microcirculation in the brain and makes the periventricular white matter particularly vulnerable to hypoxic-ischemic damage and, consequently, susceptible to the development of cPVL (34).

In our study, FIP/SIP was found to be a significant parameter in preterm infants with cPVL: 24% of cases with cPVL, but none in the control group. In a large population-based cohort study, Shah et al. (35) also showed that intestinal perforations in very preterm infants were associated with a significantly higher likelihood of severe complications including WMI. The pathophysiology of association between FIP/SIP and cPVL is not fully understood, but FIP/SIP is associated with requirement of invasive ventilation, surgery and inflammatory reactions that may impair cerebral perfusion. It is interesting to note that NEC was not associated with an increased risk of cPVL in our cohort. This may appear counterintuitive, as NEC is typically accompanied by a pronounced inflammatory response. One possible explanation could be related to the timing of onset: in very premature infants, FIP/SIP usually occurs in the immediate postnatal period, whereas NEC typically develops later, at a stage when neuron maturation and cerebral vascular autoregulation are more advanced. Thus, the inflammatory impact of NEC may coincide with a relatively more “stable” phase of brain development, whereas FIP/SIP occurs at an earlier, more vulnerable stage, which may explain the observed association between FIP/SIP and cPVL.

Our analysis also showed significantly higher incidence of ROP in children with cPVL compared to the control group. Impaired retinal vascular development due to hyperoxia and hypoxia-induced neovascularization plays the central role in development of ROP (36, 37). Although there is no clear causal link between ROP and cPVL, Holmström (38) has shown that visual dysfunctions occur significantly more frequently in children with cPVL. This may also be related to the fact that children with severe disease often receive more oxygen for other reasons, which both provokes the development of ROP and increases the risk of developing cPVL.

### 4.2 Association of cPVL with regularly measured and mathematically calculated parameters

In addition to infant diagnoses, we identified 24 regularly measured and mathematically calculated parameters (groups G4 and G5) that differed considerably in preterm infants with and without cPVL (Table 7).

The duration of invasive mechanical ventilation was a significant parameter in our study. There is also evidence in the literature that a longer duration of ventilation is considered as an independent risk factor for the development of cPVL. Choi et al. (39) showed that duration of 15–28 days was significantly associated with an increased incidence of cPVL, BPD and ROP. Menshykova and Dobryanskyy (40) also observed similar correlations. In their analysis, invasive ventilation of more than 60.5 h was associated with a significantly increased risk of cPVL. These results illustrate that mechanical ventilation is not only a consequence of serious underlying diseases, but may also contribute to pathophysiological processes increasing the risk of cerebral injury. There is also pathophysiological correlations between the duration of mechanical ventilation and the occurrence of IVH, which in our study was significantly associated with cPVL. Thus, ventilation-induced volume and pressure fluctuations can lead to rupture of the particularly fragile vascular structures in the germinal matrix (33).

It is important to note that numerically calculated CBF and its fluctuations showed a significant correlation with cPVL. Licht et al. (41) found that newborns with severe congenital heart defects had significantly reduced preoperative CBF associated with an increased rate of WMI. Our results also suggest that not only reduced CBF, but above all strong fluctuations in CBF could have a decisive influence on the development of cPVL. The difference (CBF—CBF_mean_), used in our analyses as independent parameter, reflects the intraindividual variation of CBF and indicates unstable cerebral perfusion conditions. These can be explained by immature cerebrovascular autoregulation with active and passive direct CBF reaction to systemic blood pressure changes. This increases the risk of abrupt fluctuations in blood flow and thus of ischemia-related brain lesions such as cPVL (3). Numerically calculated PtO_2_ also has a significant correlation with cPVL, demonstrating lower mean values for the affected group compared to the control group. Our results highlight the importance of these three calculated parameters in predicting cPVL risk, particularly because they are not routinely measured during standard neonatal monitoring.

Parameter pO₂ reflects oxygenation capacity in preterm infants with immature lungs. After birth, pO₂ averages 19.0 ± 7.9 mmHg in umbilical artery blood (42), and in stable infants <29 weeks, values of 30–70 mmHg correspond to SpO_2_ 85%–95% (43). In this study, pO₂ was below 60 mmHg in both groups and significantly lower in the affected group than in control one, indicating limited oxygenation. Mild hypoxia and the higher prevalence of RDS in cPVL infants suggest a link between impaired oxygenation and cPVL risk. Also, mean SpO₂ value is significantly lower in the affected group than in the control one though both were above the critical threshold of 80% recommended for the early postnatal period (44, 45). The slightly lower values observed in cPVL patients may suggest impaired oxygen homeostasis. Overall, our findings emphasize the importance of close monitoring of SpO₂ in preterm infants at risk. The respiratory rate was only moderately associated with cPVL. Mean values in both groups were within the normal range for preterm infants (50–70/min) (46). Both groups showed wide variability [(16/min; 172/min)], likely due to acute events such as tachypnea from pulmonary disease or postprandial increases (46).

Statistical analyses showed significantly higher heart rate in preterm infants with cPVL than in those without. The normal range is 120–160 bpm, with >180 bpm considered pathological (47); the cPVL group exceeded this limit, indicating stress. Blood pressure in preterm infants depends on gestational age, with MAD around 30 mmHg for <30 weeks and 40 mmHg for >30 weeks (47). It is also shown that blood pressure increases in stable preterm infants during the postnatal transition. In our study, infants with cPVL had significantly lower MAD, SYS, and DIA values than controls. Although still within the recommended range, these reductions may suggest impaired cerebral perfusion, supporting that even moderate pressure decreases can promote cPVL.

It was shown that infants with cPVL had significantly higher pCO_2_ and lower pH, HCO3- and BE than those without cPVL. This suggests an acid-base imbalance, linked to poorer oxygen supply of the body. This generates numerous acidic valences, which then lead to a shift in the acid-base balance as part of the body's own buffering process. A low pH alone is not dangerous but acidosis with a below −7.0 pH and a below −12 mmol/L BE, with signs of impaired consciousness or seizures, indicates asphyxia. This increases the risk of brain damage, including cPVL. Our results emphasis the importance of early blood gas testing in preterm infants, to better assess neurological risk.

In addition, Hb and Ht levels were significantly altered in infants with cPVL. Mean Hb values were slightly above those reported by Maier et al. (48) for preterm infants between 22 and 35 WG, while Ht values were within the expected range (39%–45%). However, the wide range of values indicated relevant outliers. Infants with cPVL showed significantly lower mean Hb and Ht values than those without. Jopling et al. (49) found that Hb and Ht increase with gestational age but decrease postnatally, especially in immature infants. Our results suggest that lower Hb and Ht may contribute to cPVL onset, possibly through reduced cerebral oxygen supply affecting periventricular tissue.

Preterm infants with cPVL had higher blood glucose levels than those without cPVL. This difference may indicate increased metabolic stress, even if below the hyperglycaemia threshold of 150 mg/dL (50). Hyperglycaemia in newborns can result from sepsis, cerebral haemorrhage, excessive glucose intake, or medications like corticosteroids or catecholamines, which are associated with severe neurological disorders like cPVL (50). A very important aspect is the immaturity of insulin regulation, including both insufficient insulin supply and immature insulin receptors. Hyperglycemia may therefore occur even in the absence of other relevant morbidities, but it can still influence the development of cPVL (51). Even mild disturbances in glucose homeostasis may contribute to cPVL. As the perinatal brain relies on continuous oxygen and glucose supply, brief interruptions can impair neuronal metabolism and cause structural damage (52). Elevated glucose may thus reflect metabolic imbalance, stress, or inflammation, which may exacerbate oxidative and cellular injury.

The next group of significant parameters in respect to PVL is the electrolyte (potassium, calcium, and sodium). We compared the results with the reference ranges according to Witt (53). Although his study did not refer explicitly to preterm infants, but to newborns under ten days (calcium) or under one month (potassium) of age, they provide a clinically oriented basis for comparison. The mean potassium level in both groups were within the normal range for newborns under one month (3.6–6.0 mmol/L 53). However, the difference between the groups was statistically significant. Moreover, the range in both groups included both hypo- and hyperkalemic values.

When talking about calcium, it is important to emphasize the distinction between ionized and total serum calcium. The ionized calcium measured by the BGA is the central value that the body constantly tries to maintain at a stable level and which is clinically important for cardiac activity and muscular-vascular influence. Serum calcium is controlled by the interaction between food intake and phosphate. Calcium levels in both groups were below the newborn reference range (1.8–2.6 mmol/L) (53), with significant group differences and marked individual variation. Hypocalcemia was more frequent in the control group, possibly reflecting immature calcium regulation in very preterm infants or perinatal asphyxia, a known cPVL risk factor. It often follows birth in asphyxiated newborns due to systemic hypoxia and interrupted placental calcium transfer (54), potentially causing neuromuscular instability and cerebral perfusion disturbances.

Perrott et al. (55)[ identified hypernatremia as a risk factor for severe neurological impairment in very preterm infants. In our analysis, no statistically significant difference in mean sodium levels was observed between children with and without cPVL, though *p* = 0.0535 suggests a possible trend. This may indicate a link between sodium balance and neurological outcome. However, the slightly higher sodium in the cPVL group could also reflect normal physiological fluctuations.

Preterm infants with cPVL had significantly lower average thrombocyte counts than those without cPVL. Thrombocyte are crucial for blood clotting, and thrombocytopenia is common in very preterm infants. In our study, counts in both groups remained within the normal range (150−450 10^9^/L) (56), but cPVL infants had significantly lower values.

In the study cohort, preterm infants with cPVL had significantly lower lymphocyte counts than those without cPVL. Since infections are often associated with lymphopenia, the significantly lower lymphocyte count in preterm infants with cPVL may indicate an inflammatory response in the pathogenesis of cPVL. However, there was no significant difference in total leukocyte count between groups. This result partly confirms the findings of Paul et al. (57), who were unable to establish a significant correlation between cPVL and changes in the peripheral blood count.

Our data confirm previous findings of significantly elevated CRP levels in preterm infants with cPVL compared to the control group (58). In addition, Cohen-Eilig et al. demonstrated a correlation between elevated CRP levels and maximum inspiratory oxygen demand in the first 12 h of life indicating an inflammatory response triggered by hypoxia or hyperoxia. Such fluctuations in oxygen supply have been shown to activate inflammatory signaling pathways that lead to damage of vulnerable cell populations in the white matter (59).

As a limitation of our study, we should note that continuous monitoring of clinical data was not available at the time of data collection, so they could not be included in the study. The reason is that present study was a retrospective one, based on data collected from two hospitals during the survey period. Future studies will focus on the multivariable pathogenesis of cPVL. Based on this, we plan to analyze what happens when more than one of the studied variables is associated with the diagnosis of cPVL. Furthermore, we will use multivariate regression methods to identify the key factors taking into account all the variables investigated. This approach could form the basis for cPVL predictive models.

## 5 Conclusion

This study compared preterm infants with and without cPVL regarding prenatal diagnoses, birth related parameters, infant diagnoses, regular measured and numerically calculated parameters (CBF, CBF calculations and PtO_2_). Four infant diagnoses (IVH, RDS, FIP/SIP, ROP) and 24 routine parameters differed considerably between affected and control groups. More over, unlike Abiramalatha et al. (60), who found limited predictive parameters, our data reveal early measurable changes preceding cPVL. These included duration of mechanical intubation, blood gas parameters, pressure, heart rate, oxygen, hematologic, inflammatory, metabolic, and cerebral perfusion markers. Notably that numerically calculated CBF, CBF fluctuations, and PtO₂ were significantly associated with cPVL. These routinely recorded parameters may support early cPVL risk assessment and deepen understanding of its pathophysiology.

## Data Availability

The raw data supporting the conclusions of this article will be made available to any qualified researcher upon request to the corresponding author. Requests to access these datasets should be directed to renee.lampe@tum.de.

## References

[1] Marlow N Ni Y Lancaster R Suonpera E Bernardi M Fahy A No change in neurodevelopment at 11 years after extremely preterm birth. Arch Dis Child Fetal Neonatal Ed. (2021) 106:418–24. 10.1136/archdischild-2020-320650 33504573

[2] Volpe JJ. Dysmaturation of premature brain: importance, cellular mechanisms, and potential interventions. Pediatr Neurol. (2019) 95:42–66. 10.1016/j.pediatrneurol.2019.02.016 30975474

[3] Volpe JJ. Neurobiology of periventricular leukomalacia in the premature infant. Pediatr Res. (2001) 50:553–62. 10.1203/00006450-200111000-00003 11641446

[4] Back SA. White matter injury in the preterm infant: pathology and mechanisms. Acta Neuropathol. (2017) 134:331–49. 10.1007/s00401-017-1718-6 28534077 PMC5973818

[5] Volpe JJ. Brain injury in the premature infant–from pathogenesis to prevention. Brain Dev. (1997) 19:519–34. 10.1016/s0387-7604(97)00078-8 9440796

[6] Hatzidaki E Giahnakis E Maraka S Korakaki E Manoura A Saitakis E Risk factors for periventricular leukomalacia. Acta Obstet Gynecol Scand. (2009) 88:110–5. 10.1080/00016340802503039 18951221

[7] Romero-Guzman GJ Lopez-Munoz F. Prevalencia y factores de riesgo de leucomalacia periventricular en recien nacidos prematuros. Revision sistematica [prevalence and risk factors for periventricular leukomalacia in preterm infants. A systematic review. Rev Neurol. (2017) 65:57–62. 10.33588/rn.6502.2017002 28675256

[8] Collins MP Lorenz JM Jetton JR Paneth N. Hypocapnia and other ventilation-related risk factors for cerebral palsy in low birth weight infants. Pediatr Res. (2001) 50:712–9. 10.1203/00006450-200112000-00014 11726729

[9] Altman DI Powers WJ Perlman JM Herscovitch P Volpe SL Volpe JJ. Cerebral blood flow requirement for brain viability in newborn infants is lower than in adults. Ann Neurol. (1988) 24:218–26. 10.1002/ana.410240208 3263081

[10] Lampe R Botkin N Turova V Blumenstein T Alves-Pinto A. Mathematical modelling of cerebral blood circulation and cerebral autoregulation: towards preventing intracranial hemorrhages in preterm newborns. Comput Math Methods Med. (2014) 2014:965275. 10.1155/2014/965275 25126111 PMC4122005

[11] Sidorenko I Turova V Botkin N Eckardt L Alves-Pinto A Felderhoff-Müser U Modeling cerebral blood flow dependence on carbon dioxide and mean arterial blood pressure in the immature brain with accounting for the germinal matrix. Front Neurol. (2018) 9:812. 10.3389/fneur.2018.00812 30356709 PMC6189337

[12] Sidorenko I Kovtanyuk A Feddahi N Jahn S Lück E Brickmann C Numerical estimation of optimal limits of cerebral blood flow and oxygen partial pressure in brain tissue of preterm infants. Comput Methods Programs Biomed. (2025) 270:108984. 10.1016/j.cmpb.2025.108984 40714416

[13] Kovtanyuk A Chebotarev A Lampe R. Mathematical modeling of cerebral oxygen transport from capillaries to tissue. Front Appl Math Stat. (2023) 9:1257066. 10.3389/fams.2023.1257066

[14] Agut T Alarcon A Cabañas F Bartocci M Martinez-Biarge M Horsch S; eurUS.brain group. Preterm white matter injury: ultrasound diagnosis and classification. Pediatr Res. (2020) 87(Suppl 1):37–49. 10.1038/s41390-020-0781-1 32218534 PMC7098888

[15] Dewan MV Herrmann R Schweiger B Sirin S Müller H Storbeck T Are simple magnetic resonance imaging biomarkers predictive of neurodevelopmental outcome at two years in very preterm infants? Neonatology. (2019) 116:331–40. 10.1159/000501799 31454812

[16] Turova V Sidorenko I Eckardt L Rieger-Fackeldey E Felderhoff-Müser U Alves-Pinto A Machine learning models for identifying preterm infants at risk of cerebral hemorrhage. PLoS One. (2020) 15(1):e0227419. 10.1371/journal.pone.0227419 31940391 PMC6961932

[17] Sidorenko I Brodkorb S Felderhoff-Müser U Rieger-Fackeldey E Krüger M Feddahi N Assessment of intraventricular hemorrhage risk in preterm infants using mathematically simulated cerebral blood flow. Front Neurol. (2024) 15:1465440. 10.3389/fneur.2024.1465440 39494169 PMC11527722

[18] Piechnik SK Chiarelli PA Jezzard P. Modelling vascular reactivity to investigate the basis of the relationship between cerebral blood volume and flow under CO2 manipulation. Neuroimage. (2008) 39:107–18. 10.1016/j.neuroimage.2007.08.022 17920935

[19] Kinoshita Y Okudera T Tsuru E Yokota A. Volumetric analysis of the germinal matrix and lateral ventricles performed using MR images of postmortem fetuses. AJNR Am J Neuroradiol. (2001) 22:382–8. PMID: 11156787 PMC7973932

[20] Urlesberger B Müller W Ritschl E Reiterer F. The influence of head position on the intracranial pressure in preterm infants with posthemorrhagic hydrocephalus. Childs Nerv Syst. (1991) 7:85–7. 10.1007/BF00247862 1863934

[21] Easa D Tran A Bingham W. Noninvasive intracranial pressure measurement in the newborn: an alternate method. Am J Dis Child. (1983) 137:332–5. 10.1001/archpedi.1983.02140300014004 6829512

[22] Sidorenko I Turova VL Kovtanyuk AE Lampe R. The numerical assessment of cerebral blood flow in immature brain of preterm infants. MMSC 2020 (2020). p. 199–207. Available online at: http://ceur-ws.org/Vol-2783/paper15.pdf (Accessed March 31, 2026).

[23] Botkin ND Kovtanyuk AE Turova VL Sidorenko IN Lampe R. Accounting for tube hematocrit in modeling of blood flow in cerebral capillary networks. Comput Math Methods Med. (2019) 2019:4235937. 10.1155/2019/4235937 31531122 PMC6721022

[24] Sidorenko I Turova V Rieger-Fackeldey E Felderhoff-Müser U Kovtanyuk A Brodkorb S Mathematical modeling of the hematocrit influence on cerebral blood flow in preterm infants. PLoS One. (2021) 16:e0261819. 10.1371/journal.pone.0261819 34962951 PMC8714087

[25] Hoffmann KH Marx D Botkin ND. Drag on spheres in micropolar fluids with non-zero boundary conditions for microrotations. J Fluid Mech. (2007) 590:319–30. 10.1017/S0022112007008099

[26] Krogh A. The number and distribution of capillaries in muscles with calculations of the oxygen pressure head necessary for supplying the tissue. J Physiol. (1919) 52:409–15. 10.1113/jphysiol.1919.sp001839 16993405 PMC1402716

[27] Wenger RH Kurtcuoglu V Scholz CC Marti HH Hoogewijs D. Frequently asked questions in hypoxia research. Hypoxia (Auckl). (2015) 3:35–43. 10.2147/HP.S92198 27774480 PMC5045069

[28] Kovtanyuk A Sidorenko I Park N Lampe R. Interactive software for simulation of oxygen transport from capillaries to brain tissue of preterm infants. EPiC Series Comput. (2024) 104:175–84.

[29] Jung A Faltermeier R Rothoerl R Brawanski A. A mathematical model of cerebral circulation and oxygen supply. J Math Biol. (2005) 51:491–507. 10.1007/s00285-005-0343-5 16195926

[30] Gursoy T Hayran M Derin H Ovali F. A clinical scoring system to predict the development of bronchopulmonary dysplasia. Am J Perinatol. (2015) 32:659–66. 10.1055/s-0034-1393935 25285400

[31] Ballabh P. Intraventricular hemorrhage in premature infants: mechanism of disease. Pediatr Res. (2010) 67:1–8. 10.1203/PDR.0b013e3181c1b176 19816235 PMC2799187

[32] Grant EG Schellinger D Smith Y Uscinski RH. Periventricular leukomalacia in combination with intraventricular hemorrhage: sonographic features and sequelae. AJNR Am J Neuroradiol. (1986) 7:443–7. PMID: 3085449 PMC8331337

[33] Volpe JJ. Neurology of the Newborn. 5th ed. Philadelphia: Saunders Elsevier (2008).

[34] Bergann A Mühler M Chaoui R Heling K Kalache K Bollmann R. Fetale leukomalazie. Ultraschall Med. (2006) 27(S1):P_3_7. 10.1055/s-2006-953892

[35] Shah J Singhal N da Silva O Rouvinez-Bouali N Seshia M Lee SK Intestinal perforation in very preterm neonates: risk factors and outcomes. J Perinatol. (2015) 35:595–600. 10.1038/jp.2015.41 25927271

[36] Smith LE Hard AL Hellström A. The biology of retinopathy of prematurity: how knowledge of pathogenesis guides treatment. Clin Perinatol. (2013) 40:201–14. 10.1016/j.clp.2013.02.002 23719305 PMC3673697

[37] Rashidian P Karami S Salehi SA. A review on retinopathy of prematurity. Med Hypothesis Discov Innov Ophthalmol. (2025) 13:201–12. 10.51329/mehdiophthal1511 40065804 PMC11890260

[38] Petri S Tinelli F. Visual impairment and periventricular leukomalacia in children: a systematic review. Res Dev Disabil. (2023) 135:104439. 10.1016/j.ridd.2023.104439 36796269

[39] Choi YB Lee J Park J Jun YH. Impact of prolonged mechanical ventilation in very low birth weight infants: results from a national cohort study. J Pediatr. (2018) 194:34–39.e3. 10.1016/j.jpeds.2017.10.042 29198532

[40] Menshykova AO Dobryanskyy DO. Duration of mechanical ventilation and clinical outcomes in very low birth weight infants: a single center 10-years cohort study. J Neonatal Perinatal Med. (2023) 16:673–80. 10.3233/NPM-230142 38043024

[41] Licht DJ Wang J Silvestre DW Nicolson SC Montenegro LM Wernovsky G Preoperative cerebral blood flow is diminished in neonates with severe congenital heart defects. J Thorac Cardiovasc Surg. (2004) 128:841–9. 10.1016/j.jtcvs.2004.07.022 15573068

[42] Dickinson JE Eriksen NL Meyer BA Parisi VM. The effect of preterm birth on umbilical cord blood gases. Obstet Gynecol. (1992) 79:575–8. PMID: 1553180

[43] Quine D Stenson BJ. Arterial oxygen tension (Pao2) values in infants <29 weeks of gestation at currently targeted saturations. Arch Dis Child Fetal Neonatal Ed. (2009) 94:F51–3. 10.1136/adc.2007.135285 18285372

[44] Dawson JA Kamlin CO Vento M Wong C Cole TJ Donath SM Defining the reference range for oxygen saturation for infants after birth. Pediatrics. (2010) 125:e1340–7. 10.1542/peds.2009-1510 20439604

[45] Sweet DG Carnielli V Greisen G Hallman M Ozek E Pas T European Consensus guidelines on the management of respiratory distress syndrome—2019 update. Neonatology. (2019) 115:432–50. 10.1159/000499361 30974433 PMC6604659

[46] Larsen R. Pädiatrische intensivmedizin. In: Reinhard L Tobias F, editors. Anästhesie und Intensivmedizin für die Fachpflege. Berlin, Heidelberg: Springer (2016). p. 920–49. 10.1007/978-3-662-50444-4_64

[47] Jorch G Hübler M. Neonatologie (2. Aufl.). Thieme (2010). Available online at: https://www.thieme-connect.de/products/ebooks/book/10.1055/b-0034-88741 (Accessed March 31, 2026).

[48] Maier RF. Anämien des früh- und neugeborenen. In: Gadner H Gaedicke G Niemeyer C Ritter J, editors. Pädiatrische Hämatologie und Onkologie. Berlin. Heidelberg: Springer (2006). p. 278–84. 10.1007/3-540-29036-2_26

[49] Jopling J Henry E Wiedmeier SE Christensen RD. Reference ranges for hematocrit and blood hemoglobin concentration during the neonatal period: data from a multihospital health care system. Pediatrics. (2009) 123:e333–7. 10.1542/peds.2008-2654 19171584

[50] Teising D Jipp H Teising D. Versorgung von früh- und neugeborenen. In: Hannah T Nadja K, editors. Neonatologische und Pädiatrische Intensiv- und Anästhesiepflege: Praxisleitfaden. Berlin, Heidelberg: Springer (2016). p. 245–72. 10.1007/978-3-662-62902-4

[51] Ramel S Rao R. Hyperglycemia in extremely preterm infants. Neoreviews. (2020) 21:e89–97. 10.1542/neo.21-2-e89 32005719

[52] Jensen A Garnier Y Middelanis J Berger R. Perinatal brain damage–from pathophysiology to prevention. Eur J Obstet Gynecol Reprod Biol. (2003) 110(Suppl 1):S70–9. 10.1016/s0301-2115(03)00175-1 12965093

[53] Witt H. Normwerte. In: Burkhard R Klaus-Peter Z, editors. Pädiatrische Gastroenterologie. Hepatologie und Ernährung. Berlin, Heidelberg: Springer (2013). p. 827–50. 10.1007/978-3-642-24710-1_54

[54] Rai S Bhatiyani KK Kaur S. Effect of birth asphyxia on serum calcium and glucose level: a prospective study. Int J Sci Study. (2015) 3:3–6. 10.17354/ijss/2015/439

[55] Perrott S Dodds L Vincer M. A population-based study of prognostic factors related to major disability in very preterm survivors. J Perinatol. (2003) 23:111–6. 10.1038/sj.jp.7210867 12673259

[56] Curley A Stanworth SJ Willoughby K Fustolo-Gunnink SF Venkatesh V Hudson C randomized trial of platelet-transfusion thresholds in neonates. N Engl J Med. (2019) 380:242–51. 10.1056/NEJMoa1807320 30387697

[57] Paul DA Leef KH Stefano JL. Increased leukocytes in infants with intraventricular hemorrhage. Pediatr Neurol. (2000) 22:194–9. 10.1016/s0887-8994(99)00155-1 10734249

[58] Cohen-Eilig M Bar Lis N Livneh A Bassan H. Early neonatal C-reactive protein levels and periventricular leukomalacia. Isr Med Assoc J. (2021) 23:620–24. PMID: 34672442

[59] Hagberg H Mallard C Ferriero DM Vannucci SJ Levison SW Vexler ZS The role of inflammation in perinatal brain injury. Nat Rev Neurol. (2015) 11:192–208. 10.1038/nrneurol.2015.13 25686754 PMC4664161

[60] Abiramalatha T Bandyopadhyay T Ramaswamy VV Shaik NB Thanigainathan S Pullattayil AK Amboiram P. Risk factors for periventricular leukomalacia in preterm infants: a systematic review, meta-analysis, and GRADE-based assessment of certainty of evidence. Pediatr Neurol (2021) 124:51–71. 10.1016/j.pediatrneurol.2021.08.003 34537463

